# Significant role of some miRNAs as biomarkers for the degree of obesity

**DOI:** 10.1186/s43141-023-00559-w

**Published:** 2023-11-06

**Authors:** Weaam Gouda, Amr E. Ahmed, Lamiaa Mageed, Amgad K. Hassan, Mie Afify, W. I. Hamimy, Halla M. Ragab, Nabila Abd El Maksoud, Abdou K. Allayeh, Mohamed D. E. Abdelmaksoud

**Affiliations:** 1 Biochemistry Department, Biotechnology Research Institute, National Research Centre, Giza, Egypt; 2 Department of Biotechnology and Life Science, Faculty of Postgraduate Studies for Advanced Sciences, Beni-Suef University, Beni-Suef, Egypt; 3 Anesthesia Department, Obesity, Surgery Unit, Faculty of Medicine, Cairo University, Giza, Egypt; 4 Environment and Climate Change Institute, National Research Centre, Giza, Egypt

**Keywords:** Obesity, MicroRNAs, Body mass index (BMI), Obesity classes, Quantitative real-time PCR (RT-qPCR)

## Abstract

**Background:**

Obesity is one of the most serious problems over the world. MicroRNAs have developed as main mediators of metabolic processes, playing significant roles in physiological processes. Thus, the present study aimed to evaluate the expressions of (miR-15a, miR-Let7, miR-344, and miR-365) and its relationship with the different classes in obese patients.

**Methods:**

A total of 125 individuals were enrolled in the study and classified into four groups: healthy non-obese controls (*n* = 50), obese class I (*n* = 24), obese class II (*n* = 17), and obese class III (*n* = 34) concerning body mass index (BMI < 30 kg/m^2^, BMI 30–34.9 kg/m^2^, BMI 35–39.9 kg/m^2^ and BMI ≥ 40 kg/m^2^, respectively). BMI and the biochemical measurements (fasting glucose, total cholesterol, triglycerides, HDL and LDL, urea, creatinine, AST, and ALT) were determined. The expressions of (miR-15a, miR-Let7, miR-344, and miR-365) were detected through quantitative real-time PCR (RT-qPCR).

**Results:**

There was a significant difference between different obese classes and controls (*P* < 0.05) concerning (BMI, TC, TG, HDL, and LDL). In contrast, fasting glucose, kidney, and liver functions had no significant difference. Our data revealed that the expression of miR-15a and miR-365 were significantly associated with different obese classes. But the circulating miR-Let7 and miR-344 were not significantly related to obesity in different classes.

**Conclusion:**

Our study indicated that miR-15a and miR-365 might consider as biomarkers for the obesity development into different obese classes. Thus, the relationship between regulatory microRNAs and disease has been the object of intense investigation.

## Background

Obesity is the accumulation of extra fat, leading to health complications [1]. The primary components of adipose tissue are fat cells or adipocytes, and diverse functions for adipose tissue depots have been identified in controlling the predisposition to obesity [2]. In 2017, obesity accounted for 8.4% of all mortality worldwide, ranking it fifth among the most preventable causes of death [3]. According to the World Health Organization (WHO), Egypt has the 18th-highest rate of obesity in the world [1]. Around 71% of all deaths are attributed to non-communicable diseases [4]. The classifications for BMI are in use by the National Institute of Health and the WHO: obesity-BMI higher than or equal to 30 kg/m^2^, obesity class I-BMI 30 to 34.9 kg/m^2^, obesity class II-BMI 35 to 39.9 kg/m^2^, and obesity class III-BMI higher than or equal to 40 kg/m^2^ (also referred to as severe, extreme, or massive obesity) [1].

Given its rising prevalence, the WHO also identified obesity as a significant susceptibility indicator for the emergence of chronic diseases including cancer, diabetes, and various coronary disorders [1]. As a result, obesity causes a variety of metabolic abnormalities, which harm adipose tissue and the cardiovascular system phenotypically and functionally [2]. Even so, limited data has been established concerning the genetic alterations caused by obesity and what potential cellular processes can be used to treat obesity. A number of treatments have been suggested to minimize the prevalence of obesity and alleviate the comorbidities produced by obesity [5].

MicroRNAs (miRNAs) have been considered therapeutics for many diseases, including metabolic disorders [2]. The two main functions of miRNAs (class of small non-coding RNA molecules) are post-transcriptional and RNA silencing, which regulates the expression of genes [2]. As a result, numerous attempts have been undertaken to alter the miRNA expression in order to suppress, raise, or restore their expressions for therapeutic purposes [6]. Due to their unique properties, including their highly conserved short nucleotide sequences and well-known compositions, they are a potential platform for developing new therapeutics for miRNA-associated diseases [2].

The usage of miRNAs as circulating biomarkers is an interesting tool for early detection to recognize individuals at risk of disease development and might indicate the expression of adipose tissue [7]. Additionally, numerous studies have indicated that miRNAs are involved in adipogenic process, like cardiac lipotoxicity, cardiac hypertrophy, microvascular rarefaction, atherosclerosis, skeletal muscle phenotypic changes, and other comorbidities [2 & 6]. Unfortunately, there is still little knowledge available about the potential mechanisms [8]. For instance, it has been demonstrated that the miRNAs promote adipogenesis through different mechanisms and interfere with adipocyte differentiation (like microRNA-15, microRNA-Let-7, microRNA-344, and microRNA-365) [9].

The microRNA-15 (miR-15) is a member of the vmiR-15 family, which is identified as highly preserved among species. These miRNAs are expressed in a variety of organs, including the heart, skeletal muscle, liver, kidney, brain, lung, and spleen [10]. Presently, the majority of miR-15a research focuses on human disorders, and only a small number of studies on animal fat regulation suggest that miR-15a is crucial for animal fat metabolism or adipogenesis [11, 12].

In addition, Let-7 was the first human miRNA to be identified. This miRNA is one of 11 members of a well-conserved family that is linked to numerous essential cell processes including proliferation, cell cycle checkpoints, and apoptosis. This miRNA family has a significant role in developmental processes and directly regulates oncogenes including the renin-angiotensin system (RAS) and high mobility group A2 (HMGA2) [13, 14]. Moreover, it controls the change from clonal expansion to terminal differentiation as an anti-adipogenic factor [15].

MicroRNA-344 (miR-344) was isolated initially from embryonic primary rat cortical neurons [16]. It is expressed during mouse brain development [17]. However, the function of miR-344 is so far unclear, and previous studies suggested that miR-344 may be involved in the regulation of adipocyte differentiation [18]. Furthermore, MiR-365 is found on chromosome 16p13.12 and is involved in several physiological processes, such as lung development, cell cycle progression, and apoptosis [19]. In addition, one of the mechano-responsive miRNAs, miR-365, was previously recognized for its potent ability to stimulate inflammatory signs [20]. Based on this information, the goal of our study was to evaluate the expressions of miR-15a, miR-Let7, miR-344, and miR-365 in the obese patients and to assess their potential as molecular biomarkers of obesity classes.

## Methods

A total of 125 individuals were included and classified regarding BMI into four groups: (1) healthy non-obese controls (*n* = 50; BMI < 30 kg/m^2^), (2) obese class I patients (*n* = 24; BMI 30–34.9 kg/m^2^, (3) obese class II patients (*n* = 17; BMI 35–39.9 kg/m^2^, and (4) obese class III patients (*n* = 34; BMI ≥ 40 kg/m^2^). BMI measured (weight in kilograms/height in meters squared).

Five ml of venous blood obtained from each subject after an overnight fast was divided into two tubes: the first tube with EDTA for miRNA investigations and the second tube for the blood serum that was separated by centrifugation at 3500 rpm for 15 min following 30 min of clotting at room temperature. The clear, non-hemolysed supernatant sera was stored at − 80℃ for subsequent biochemical analysis. We performed the biochemical measurements with the usual techniques of the clinical laboratory, using commercial kits following the manufacturer’s instructions. This project was approved by the Ethics Committee of the National Research Centre (No. 19–162) that was conformed to the provisions of the Declaration of Helsinki, and a written informed consent was obtained from all volunteers.

The expressions of miRNAs (15a, Let-7, 344, and 365) were performed in blood samples for obese and non-obese groups. The total RNAs were extracted from plasma using the TRIzol reagent (Qiagen). RNA samples (1 μg) were reverse transcribed using TaqMan RT reagents from Qiagen. A SYBR Premix Ex Taq II (Applied Biosystems) was used to carry out quantitative real-time PCR. RT-qPCR reactions were performed using a PCR system 2700 real-time PCR machine (Applied Biosystems, USA). Ninety-five degrees Celsius for 1 min, followed by 40 cycles of 95℃ for 15 s, 55℃ for 30 s, and 72℃ for 30 s, were the reaction conditions for PCR. U6 served as a miRNA endogenous control, and each reaction was done in triplicate. Relative expressions of different miRNAs were evaluated through the 2^−ΔΔCt^ method [21].

### Statistical analysis

Our study used SPSS 22.0 (IBM, USA) for statistical analysis. Data were represented as mean ± standard error (SE). One-way analysis of variance (ANOVA) was used to analyze differences within or between groups. Additionally, Pearson’s correlation analysis was used to determine the correlation coefficient (R) of the expression level between miRNAs and parameters. *P* value is considered statistically significant at *P* < 0.05. The significant correlations were represented as scatterplot graphs.

## Results

### Anthropometric measurements and clinical data of obese patients and controls

The study comprised 75 obese patients, who were classified according to BMI into different obesity classes:

- Class I: BMI with a mean of 33 kg/m^2^ their age ranges from 28 to 51 with a mean of 40 years (No. = 24).
- Class II: BMI with a mean of 37 kg/m^2^ their age ranges from 30 to 48 with a mean of 39 years (No. = 17).
- Class III: with a mean of 47 kg/m^2^ their age ranges from 24 to 57 with a mean of 42 years (No. = 34).

Beside 50 healthy normal weight controls their age was 40 ± 0.93 years, and the mean BMI was 21.14 ± 0.36 kg/m^2^. The laboratory data of obese patients and controls concerning (fasting glucose; lipid profile total cholesterol, triglycerides, HDL-cholesterol, and LDL-cholesterol; kidney functions urea and creatinine; liver functions AST and ALT) are shown in Table 1. There was a statistically significant difference (*P* < 0.05) between different obese classes and controls according to BMI, TC, TG, HDL-C, and LDL-C) while fasting glucose level, kidney functions, and liver functions showed non-significant difference.

**Table 1:** Anthropometric measurements and laboratory data of obese patients and controls

Parameter	Obese (n = 75)			Controls (*n* = 50)	*P* value
	**Obese class III (*n* = 34)**	**Obese class II (*n* = 17)**	**Obese class I (*n* = 24)**		
**Age (years)**	42± 2.2	39 ± 3.7	40 ± 2.4	40 ± .0.93	0.874
**Sex, *n* (%)**					
**Male**	13 (38.2%)	6 (35.3%)	9 (37.5%)	28 (56%)	0.233
**Female**	21 (61.8%)	11 (64.7%)	15 (62.5%)	22 (44%)	
**Body mass index (kg/m**^**2**^**)**	43 ± 0.8	37 ± 0.2	33 ± 0.4	21.14 ± 0.36	**0.000**
**Fasting glucose (mg/dL)**	87 ± 3	86 ± 3.5	84 ± 2.3	90 ± 1.4	0.222
**Total cholesterol (mg/dL)**	189 ± 1.5	187 ± 1.3	154 ± 1.8	145.8 ± 1.6	**0.000**
**Triglycerides (mg/dL)**	139 ± 3.1	130 ± 1.7	109 ± 1.5	87.9 ± 2	**0.000**
**HDL-cholesterol (mg/dL)**	55 ± 4	60.7 ± 1.8	59.5 ± 0.7	50 ± 0.5	**0.000**
**LDL-cholesterol (mg/dL)**	104 ± 0.39	100 ± 0.36	108 ± 0.3	77.7 ± 0.6	**0.001**
**Urea (mg/dL)**	24 ± 0.9	25.2 ± 0.92	22.6 ± 0.96	23.14 ± 0.4	0.298
**Creatinine (mg/dL)**	0.91 ± 0.05	0.92 ± 0.06	0.93 ± 0.03	0.89 ± 0.02	0.682
**AST (IU/L)**	22 ± 1.5	24 ± 1.4	24 ± 1.1	23 ± 0.4	0.504
**ALT (IU/L)**	23.6 ± 1.5	23.4 ± 0.6	24.5 ± 0.7	24 ± 0.5	0.876

Numeric variables are presented as mean ± SE*. P* value for comparison between obese and control groups. *P* value < 0.05 are represented in bold font and considered as statistically significant

*HDL* High-density lipoprotein, *LDL* Low-density lipoprotein, *AST* Aspartate aminotransferase, *ALT* Alanine aminotransferase

### The expression of the circulating miRNAs in different obese classes and the normal weight controls

**Fig. 1:**
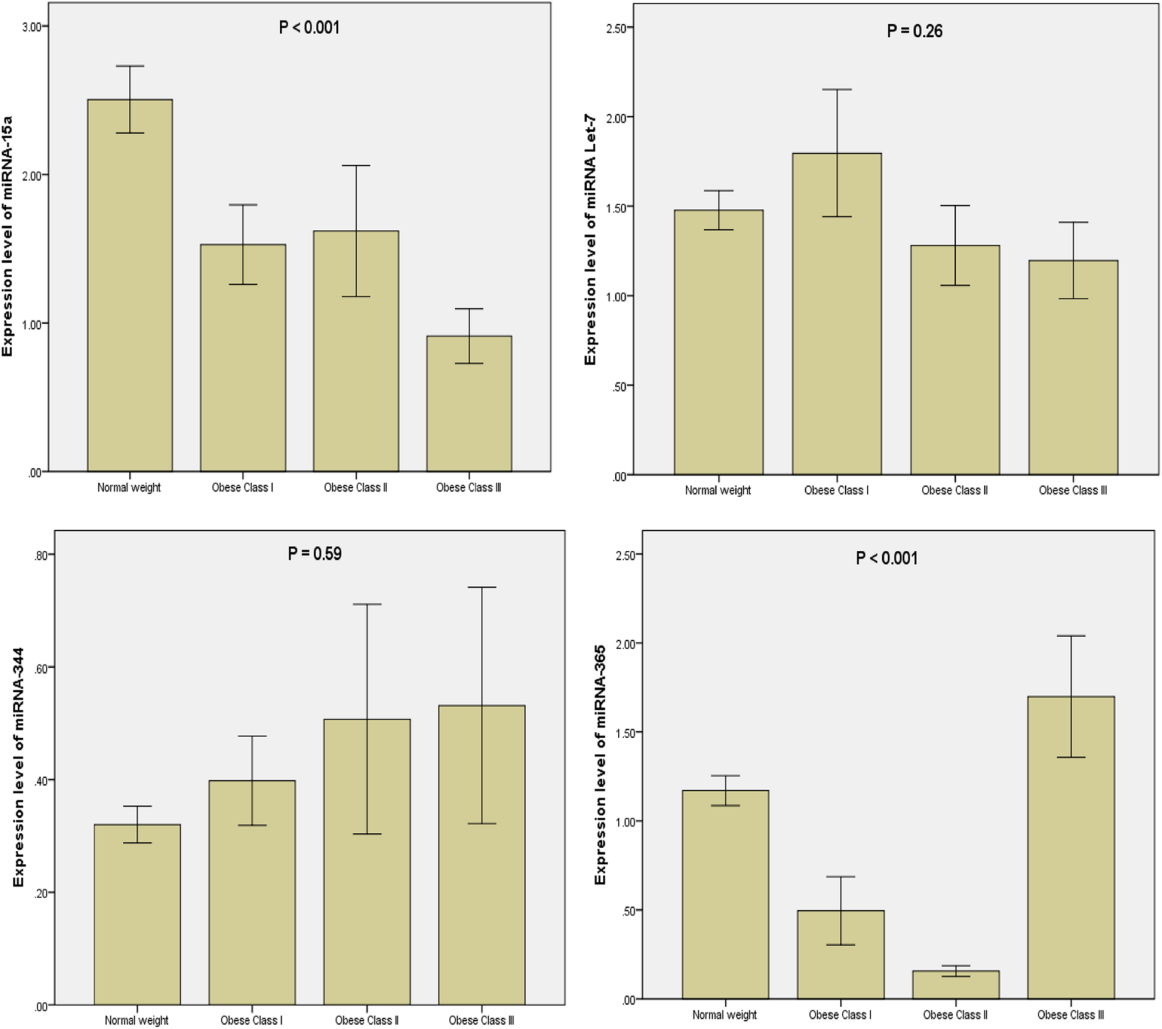
The relative miRNA expressions in obese class I, class II, class III, and control groups. Control group: Healthy subjects with normal weight BMI < 30 kg/m^2^. Obese class I group: Obese patients BMI 30–34.9 kg/m^2^. Obese class II group: obese patients BMI 35–39.9 kg/m^2^. Obese class III group: obese patients BMI ≥ 40 kg/m^2^

To identify the circulating miRNAs that were involved in the regulation of obesity, we calculated the level of the circulating miRNAs (15a, Let7, 344, and 365) within plasma samples from normal weight and obese subjects. As given in Fig. 1, circulating miR-15a and miR-365 significantly differed between different obese classes’ patients and normal weight healthy control subjects. However, the circulating miRNA-344 and Let-7 did not significantly differed between obese classes and controls.

### Coefficients of linear correlation (r) between the circulating miRNAs and BMI and lipid profile in different obese class patients

**Fig. 2:**
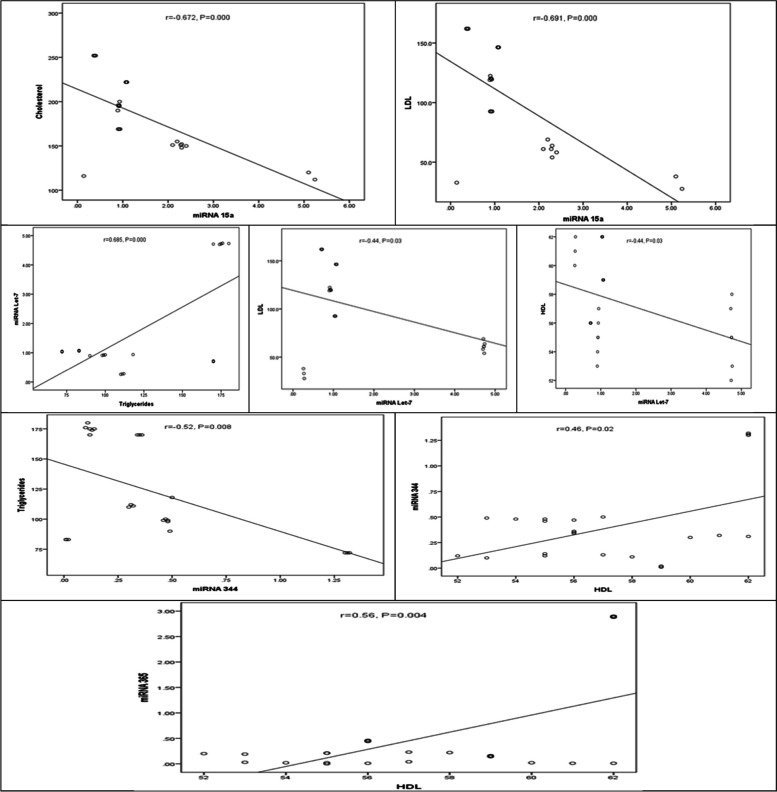
Correlations of miRNAs with lipid profile in obese class I

**Fig. 3:**
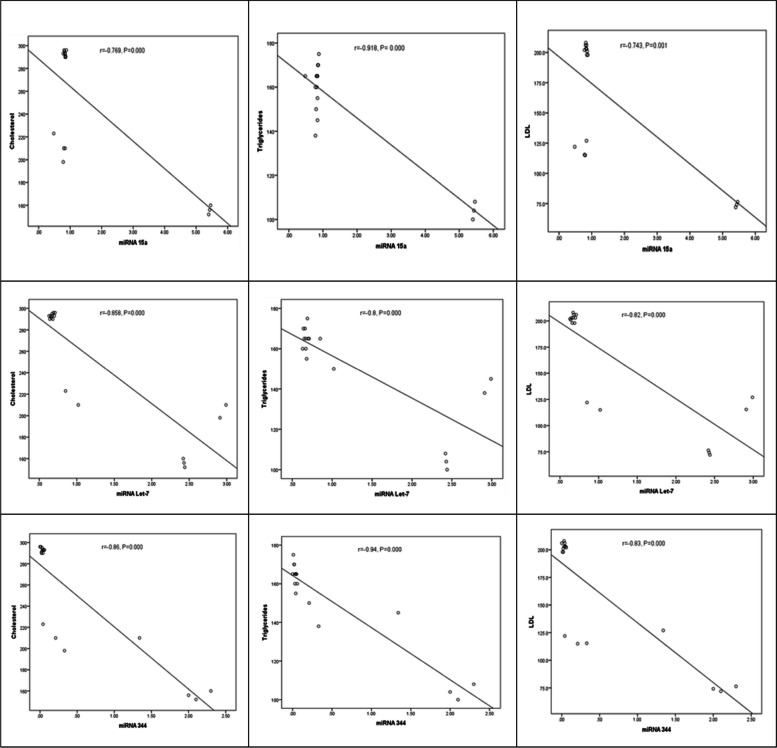
Correlations of miRNAs with lipid profile in obese class II

**Fig. 4:**
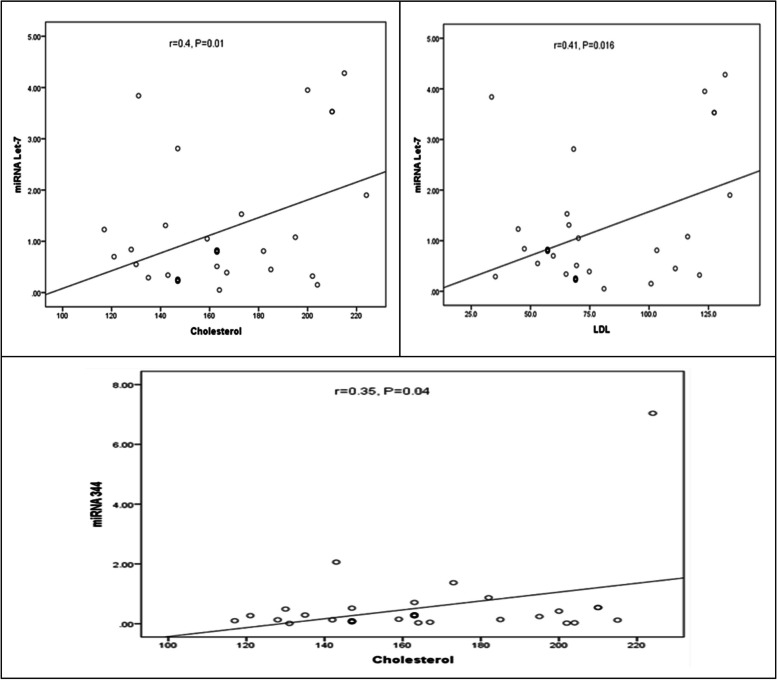
Correlations of miRNAs with lipid profile in obese class III

Tables 2, 3, and 4 and Figs. 2, 3, and 4 indicated the correlation (r) between the circulating miRNA levels and BMI and lipid profile for obese patients’ classes.

**Table 2:** Coefficients of linear correlation (*r*) between circulating miRNAs and BMI and lipid profile in obese class I patients

		**BMI**	**TC**	**TG**	**LDL**	**HDL**
**miRNA 15a**	Correlation	0.019	**-0.672**^b^	0.182	**-0.691**^b^	0.155
	Significant	0.929	**0.000**	0.395	**0.000**	0.471
**miRNA Let-7**	Correlation	**0.417**^a^	**-**0.356	**0.685**^b^	**-0.441**^a^	**-0.443**^a^
	Significant	**0.043**	0.088	**0.000**	**0.031**	**0.030**
**miRNA 344**	Correlation	**-0.436**^a^	**-**0.044	**-0.526**^b^	0.025	**0.463**^a^
	Significant	**.033**	0.838	**0.008**	0.908	**0.023**
**miRNA 365**	Correlation	**0.565**^b^	**-**0.038	**-**0.381	**-**0.004	**0.560**^b^
	Significant	**0.004**	0.860	0.066	0.985	**0.004**

*P* value < 0.05 are represented in bold font and considered as statistically significant

*BMI* Body mass index, *TC* Total cholesterol, *TG* Triglycerides, *HDL* High-density lipoprotein, *LDL* Low-density lipoprotein

^a^Correlation is significant at the 0.05 level

^b^Correlation is significant at the 0.01 level

**Table 3:** Coefficients of linear correlation (r) between circulating miRNAs and BMI and lipid profile in obese class II patients

		BMI	TC	TG	LDL	HDL
**miRNA 15a**	Correlation	-0.417	**-0.769**^b^	**-0.918**^b^	**-0.743**^b^	0.245
	Significant	0.096	**0.000**	**0.000**	**0.001**	0.343
**miRNA Let-7**	Correlation	**-0.583**^a^	**-0.858**^b^	**-0.802**^b^	**-0.825**^b^	-0.043
	Significant	**0.014**	**0.000**	**0.000**	**0.000**	0.870
**miRNA 344**	Correlation	**-0.566**^a^	**-0.867**^b^	**-0.940**^b^	**-0.838**^b^	0.163
	Significant	**0.018**	**0.000**	**0.000**	**0.000**	0.532
**miRNA 365**	Correlation	-0.342	-0.031	0.225	-0.055	0.034
	Significant	0.179	0.906	0.386	0.833	0.896

*P* value < 0.05 are represented in bold font and considered as statistically significant

*BMI* Body mass index, *TC* Total cholesterol, *TG* Triglycerides, *HDL* High-density lipoprotein, *LDL* Low-density lipoprotein

^a^Correlation is significant at the 0.05 level

^b^Correlation is significant at the 0.01 level

**Table 4:** Coefficients of linear correlation (r) between circulating miRNAs and BMI and lipid profile in obese class III patients

		BMI	TC	TG	LDL	HDL
**miRNA 15a**	Correlation	0.217	-0.065	0.152	-0.169	0.143
	Significant	0.219	0.713	0.390	0.338	0.419
**miRNA Let-7**	Correlation	0.336	**0.403**^a^	0.021	**0.411**^a^	-0.090
	Significant	0.052	**0.018**	0.906	**0.016**	0.611
**miRNA 344**	Correlation	0.278	**0.352**^a^	0.011	0.317	0.091
	Significant	0.112	**0.041**	0.952	0.067	0.609
**miRNA 365**	Correlation	-0.336	-0.070	0.289	-0.239	0.163
	Significant	0.052	0.696	0.097	0.173	0.357

*P* value < 0.05 are represented in bold font and considered as statistically significant

*BMI* Body mass index, *TC* Total cholesterol, *TG* Triglycerides, *HDL* High-density lipoprotein, *LDL* Low-density lipoprotein

^a^Correlation is significant at the 0.05 level

^b^Correlation is significant at the 0.01 level

## Discussion

Obesity is a severe public health issue, and it is a complex and polygenic condition that affects both industrialized and developing countries. Obesity is a result of interactions between hereditary, metabolic, psychological, and environmental factors [22]. MiRNAs have developed as significant regulators of lipid and glucose metabolism and are crucial in the development of obesity and obesity-related disorders by impacting the structures and functions of adipose tissue, muscle, pancreas, and liver. Knowledge of the mechanisms of action is still mostly restricted due to the ability of miRNAs to simultaneously modify various pathways and gene networks and the technological limitations of in vivo profiling [23].

Numerous studies have confirmed the role of miRNAs in obesity, even though it is unclear how these molecules work in the various degrees of obesity. Here, we discuss how miRNAs influence several types of obesity. We provide instances of how miRNAs affect the onset and progression of obesity classes. We also discuss the correlation between lipid profiles and miRNAs whenever possible [23].

We revealed a strong association between miR-15a and various degrees of obesity. This indicates that miR-15a is necessary for the metabolism of lipids [24–26]. In the same line, previous studies in mammary epithelial cells have shown that miR-15a improves fat metabolism by suppressing the expression of the LDL receptor [24], downregulating fatty acid synthase expression in mammary cells [25], controlling pre-adipocyte cell size and proliferation [27], and promoting adipogenesis [28]. Correspondingly, it also reduces the lipid metabolism markers [29]. Along with this, our study observed that over-expression of miR-15a in obese patients of various classes might lead to an increase in the accumulation of LDL, triglycerides, and cholesterol. These outcomes align with those that have been reported for pre-adipocytes [27, 28].

Our results showed that there is no statistically significant relationship between classes of obese subjects and Let-7. Adipogenesis is adversely regulated by Let-7. In adipogenesis, Let-7 is increased [30]. Let-7 generally seems to serve as an anti-adipogenic factor [14]. It might be explained as the let-7 family of miRNAs, which consists of numerous paralog genes, has been found to be one of the largest and most conserved families of miRNAs across diverse species, extending from worms to humans [31].

Recently, a family of modulators known as miRNAs has been discovered which control gene expression by altering the level of mRNA or translation [32]. Therefore, microRNAs play an important role in the regulation of genes, including the pathways that control adipocyte differentiation and function. They control the differentiation of brown adipose tissue as well as the growth of brown fat in addition to white adipose tissue. Consequently, treatments based on miRNA that target these adipocyte depots may be able to overcome obesity, insulin resistance, and malfunction of the adipose tissue [33]. Hence, the ability of miRNAs to function as either pro- or anti-adipogenesis genes has been demonstrated in several studies [34–37]. MiR-344 and its target genes may therefore have a role in the pathological development of different types of obesity. In this study, we showed that miR-344 was upregulated in the different obese classes according to BMI but not statistically significant in different obese classes. Our findings were consistent with those of Qin et al., who found that miR-344 was upregulated throughout adipogenesis [18]. However, prior studies revealed that miR-344 considerably decreased during adipogenesis under standard culture conditions, indicating that miR-344 contributes to adipocyte differentiation. It is conceivable that the regulation of adipocyte differentiation may be regulated by miR-344 [35]. This may be explained by the fact that previous studies have established the fundamental function and mechanism of miR-344 in the suppression of adipocyte differentiation through the inhibition of GSK3b at the post-transcriptional level and the activation of the transcription of downstream genes of the Wnt/b-catenin signaling pathway that reduce the expression of adipogenic genes [18].

The results of our study revealed that miR-365 was considerably expressed in obese classes, along with higher HDL levels. A previous study stated that type 2 diabetic nephropathy (T2DN) has been referred to as an obesity-related renal disease, and it has demonstrated that obesity-related fat accumulation and metabolic alterations are essential for the initiation and development of T2DN [38]. Also, a prior research by Zhao et al. [20] investigated the role of miR-365 in the nephropathy induced by the HFD/STZ treatment in rats.

Regarding the correlations between the circulating miRNAs and lipid profiles among obese classes, there was a correlation between miRNA-15a and TC & LDL; Let-7 with TG, LDL, and HDL; miR-344 with TG & HDL; miR-365 with HDL in obese class I. Concerning class II: miRNAs (15a, Let-7, and 344) were significantly related to LDL, TC, and TG. Finally, the miRNA-Let7 was linked with TC & LDL while miR-344 was related with TC within class III. As is well known, numerous research teams’ contributions have highlighted the critical role that miRNAs play in controlling lipid metabolism and cholesterol homeostasis. So, lipoprotein-carried miRNAs are evolving to be used as valuable biomarkers or functional regulators. As previously mentioned, studies have emphasized some of the most significant results linked to the monitoring of HDL-C and LDL-C via miRNA. According to these results, miRNA-targeted therapeutics could represent an advanced approach for the treatment of obesity. However, it has been discovered that prolonged administration or genetic ablation of certain miRNAs produces undesirable side effects such as dyslipidemia and obesity [39]. Larger investigations will also be required to properly explore the therapeutic and biomarker potential of miRNAs carried by lipoproteins in diseases. Briefly, the role of lipoproteins and prospective therapeutic uses of miRNA-based therapies for the prevention and management of metabolic disorders make lipoprotein transport of miRNAs an attractive research area that needs further study [40].

Furthermore, as all of the study’s samples were enrolled from clinical patients, we believe that these miRNAs may be more significant than those identified in animal models and, as a result, may be better able to illuminate the potential function of the studied miRNAs in obesity. The study’s limitations comprise the sample size was small, and the findings need to be further verified in a large patient cohort.

## Conclusion

The miRNAs that have been implicated in obesity thus far may be of vital importance in the management of obesity in various developed classes. This provides an opportunity for the application of miRNA-based therapeutics when miRNAs found in the blood circulation target numerous mRNAs, have limited toxic effects, and are well tolerated by patients. Hence, the findings of this study demonstrated that miR-15a and miR-365 may be considered as biomarkers for the progression of obesity and consequently play important roles in the development of obesity in different obese classes. Consequently, the data from the present study could provide potential biomarkers for obesity treatment in the future.

## Data Availability

The datasets used and/or analyzed during the current study are available from the corresponding author on reasonable request.

## Abbreviations

BMI: Body mass index
WHO: World Health Organization
RT-qPCR: Quantitative real-time PCR
RAS: Renin-angiotensin system
HMGA2: High mobility group A2
TC: Total cholesterol
TG: Triglycerides
HDL: High-density lipoprotein
LDL: Low-density lipoprotein
AST: Aspartate aminotransferase
ALT: Alanine aminotransferase
T2DN: Type 2 diabetic nephropathy
